# History of Circumcision

**Published:** 1892-06

**Authors:** 


*History of Circumcision.* By P. C. REMONDINO, M.D. Pp. 346. Philadelphia: F. A. DAVIS. 1891.

In a work of this sort, dealing with a subject which is inherently delicate, and which is intimately connected with the religion of a large number of our fellow-beings, we might expect that a medical man would write with dignity and refinement; or, at least, that he would avoid anything approaching to levity or buffoonery. Our readers must judge of the whole from an extract or two:

"Whenever the writer sees the poor anæmic, broken-down victim of many miscarriages, he cannot help but feel that, if the laws of the Damiantina River savages [performing artificial hypospadias] were enforced on their husbands, it would be a blessing to the poor women without materially injuring the husbands, who, in case of need of a re-establishment of the functions of procreation, might be fitted with a vulcanite plate for the occasion—something like our cleft-palate patients are supplied with a plate that enables them to articulate" (p. 59).

"The Grecian landscape and topography does not permit of much richness of romantic incidents or details, any more than the love-making of the unfortunate spider, who is devoured by his spidery Cleopatra at the end of his first sexual embrace, could furnish any incidents for one of Amelie Rives's spirited novels; so that neither minstrel nor bard have recorded the details of the first emasculating tragedy, which, from all accounts, was a kind of an Olympian Donnybrook-fair sort of paricidal-ending tragedy. Unfortunately, Homer was not there to describe the event, or we might have had a Wagnerian opera with its Plutonic music to illustrate all its incidents;" and so on (p. 82). The spelling and grammar in this quotation are Dr. Remondino's.

The work is disfigured throughout by such examples of bad taste; otherwise it is commendable, showing evidence of considerable study, and being, in the main, sound in a surgical sense.

